# Secreted miR-27a Induced by Cyclic Stretch Modulates the Proliferation of Endothelial Cells in Hypertension via GRK6

**DOI:** 10.1038/srep41058

**Published:** 2017-01-20

**Authors:** Lu Wang, Han Bao, Kai-Xuan Wang, Ping Zhang, Qing-Ping Yao, Xiao-Hu Chen, Kai Huang, Ying-Xin Qi, Zong-Lai Jiang

**Affiliations:** 1 Institute of Mechanobiology & Medical Engineering, School of Life Sciences & Biotechnology, Shanghai Jiao Tong University, Shanghai, China

## Abstract

Abnormal proliferation of endothelial cells (ECs) is important in vascular remodeling during hypertension, but the mechanisms are still unclear. In hypertensive rats caused by abdominal aortic coarctation, the expression of G-protein-coupled receptor kinase 6 (GRK6) in ECs at common carotid artery was repressed *in vivo*, and EC proliferation was increased. 15% cyclic stretch *in vitro*, which mimics the pathologically increased stretch in hypertension, repressed EC GRK6 expression via paracrine control by vascular smooth muscle cells (VSMCs). Furthermore, VSMC-derived microparticles (VSMC-MPs) were detected in the conditioned medium from VSMCs and in artery. VSMC-MPs from cells exposed to 15% cyclic stretch decreased GRK6 expression and increased EC proliferation. miR-27a was detected in VSMC-MPs and was upregulated by 15% cyclic stretch. miR-27a was transferred from VSMCs to ECs via VSMC-MPs and directly targeted on GRK6. Finally, a multi-point injection of antagomiR-27a around carotid artery decreased miR-27a expression *in vivo*, induced GRK6 expression, and reversed the abnormal EC proliferation. Pathologically elevated cyclic stretch increased the secretion of miR-27a, which was transferred from VSMCs to ECs via the VSMC-MPs, subsequently targeted GRK6, and induced EC proliferation. Locally decreasing miR-27a could be a novel therapeutic approach to attenuate the abnormal EC proliferation in hypertension.

Endothelial cells (ECs), located at the intima, are important cellular components of the vessel wall. Growing evidences have demonstrated that the abnormal proliferation of ECs has a crucial role in vascular remodeling during hypertension, which results in the destruction of vascular homeostasis, the obstruction of the vessel wall, a continued inflammatory response, and dysregulated communication between cells^1^,^2^. Vascular smooth muscle cell (VSMCs), which are adjacent to ECs *in vivo*, modulate EC functions via paracrine control^3^,^4^. In the hypertensive state, the cyclic stretch, defined as the repetitive deformation of vascular cells caused by the regular constriction and relaxation of the arterial wall with the cardiac cycle^5^, is elevated^6^ and is important in modulating the paracrine regulation of the VSMCs^7^,^8^.

G-protein-coupled receptor kinases (GRKs) contain seven members, which have multiple physiological effects in hypertension^9^,^10^. Preliminary experiments and the study by Tiruppathi *et al*. showed that GRK2, GRK5, and GRK6 were expressed predominantly in ECs^11^. Although numerous studies have demonstrated the relation between GRK2/5 and hypertension^9^,^12^, the roles of GRK6 in hypertension have not yet been fully elucidated^13^. Changes in GRK6 have been reported to participate in the functional regulation of various cells. GRK6 deficiency in mice impaired the engulfment of apoptotic cells^14^. Repression of GRK6 promoted migration of medulloblastoma cell^15^, and GRK6 expression has been related to proliferation in hepatocellular carcinoma patients^16^. Therefore, we examined the roles of GRK6 in EC proliferation during hypertension and the potential mechanobiological mechanism(s) involved in this process.

Similar to most proteins, GRKs can be regulated by altering their expression levels^17^. microRNAs (miRs) are important regulatory molecules that bind to complementary sequences in their target mRNAs through perfect or imperfect base pairing and promote direct cleavage or translational repression^18^. Although no studies have reported the regulation of GRKs by miRs, we hypothesized that this regulation is possible. In addition to primary intracellular locations of miRs, they have recently been found extracellularly and could be delivered to recipient cells, altering gene expression therein as well as the functions of target cells^19^,^20^,^21^. miRs can be secreted and protected extracellularly in several ways: RNA-binding proteins, lipoprotein complexes, and inclusion into membrane-derived vesicles including microparticles (MPs) and exosomes^22^. Secreted miRs participate in intercellular communication during many cardiovascular disorders; however, their role in abnormal EC proliferation in hypertension is still unknown.

Using a hypertensive animal model *in vivo* and a cyclic stretch application system *in vitro*, we investigated the roles of secreted miRs in intercellular communication between ECs and VSMCs in response to mechanical cyclic stretch and also determined the effects of GRK6 modulated by secreted miR-27a on the abnormal proliferation of ECs in hypertension. The results provide new insights into the mechanism by which hypertension regulates EC functions and will help to develop a novel therapeutic approach to attenuate the abnormal proliferation of ECs in hypertension.

## Results

### Hypertension decreased the expression of GRK6 but increased the proliferation of ECs through a paracrine mechanism

**Figure 1:**
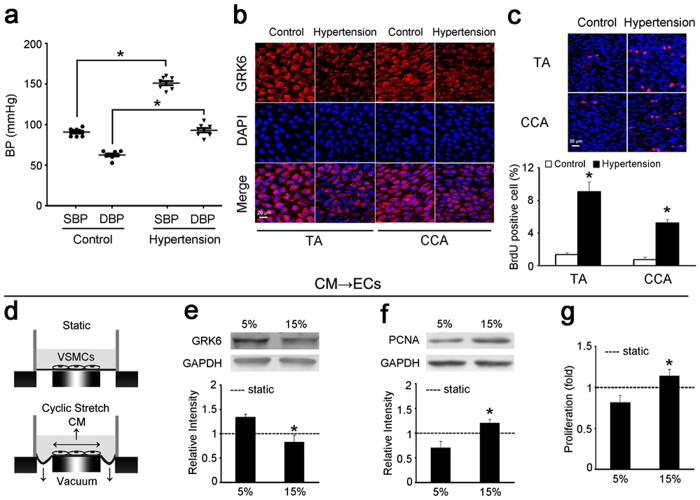
Hypertension decreased GRK6 expression but increased EC proliferation through a paracrine mechanism. (**a**) BP of 1-week hypertensive rats and normotensive controls measured through catheters introduced into the carotid arteries (n = 8). (**b**) The expression of GRK6 in the ECs of the TA and CCA from hypertensive rats and controls detected by immunofluorescence. Scale bar = 20 μm. (**c**) The proliferation of ECs in the TA and CCA from hypertensive rats and controls detected by *in situ* BrdU immunofluorescence. The histogram shows the fold change in the level of EC proliferation relative to the control. Scale bar = 20 μm. Values are shown as mean ± SD for each condition from at least four independent experiments. *P < 0.05 vs. control. (**d–g**) VSMCs were exposed to static condition, 5% and 15% cyclic stretch for 24 h, respectively, and the CM was used to stimulate ECs for 24 h. (**d**) Schematic drawing of the cyclic stretch treatment and the CM obtained. (**e**) GRK6 expression and (**f**) PCNA expression of ECs were detected by western blotting. (**g**) Proliferation of ECs was detected by BrdU ELISA. ‘----’ indicates the static values standardized to 1. The values are expressed as the mean ± SD for each condition from at least four independent experiments. *P < 0.05 vs. 5%. Full-length blots are presented in Supplementary Fig. S7.

The hypertensive rat model caused by abdominal aortic coarctation was used to detect GRK6 expression and the proliferation of ECs *in vivo*, while the sham-ligated rat was used as control. Thoracic aorta (TA) and common carotid artery (CCA) were isolated 1 week after surgery for measurements. Compared with the sham control, the blood pressure (BP) of hypertensive rats caused by abdominal aortic coarctation was markedly increased 1 week after the operation 151 ± 9 mmHg *vs.* 91 ± 6 mmHg (systolic blood pressure, SBP, *P* = 2.05 × 10^−9^) and 93 ± 9 mmHg *vs.* 63 ± 6 mmHg (diastolic blood pressure, DBP, *P* = 4.08 × 10^−6^) (Fig. 1a). The expression of GRK6 in the ECs of the TA and the CCA was significantly decreased in hypertensive rats (Fig. 1b and Supplementary Fig. S1), whereas EC proliferation was significantly increased (Fig. 1c).

Given that increased cyclic mechanical stretch in hypertension has important roles in the hyperproliferation of ECs^8^,^23^, we then investigated whether the increased cyclic stretch inhibited GRK6 expression and promoted EC proliferation. ECs were firstly exposed to different levels of cyclic stretch for 24 hours. Compared with the static, cyclic stretch (both 5% and 15%) slightly repressed GRK6 expression in ECs, but the difference was not significant. The expression of GRK6 in ECs exposed to 5% cyclic stretch (normal and physiological stretch^24^) was similar to that of 15% cyclic stretch (high and pathological stretch^6^) (Supplementary Fig. S2a). The results suggested that mechanical stretch does not directly affect GRK6 expression in ECs. Given that ECs are also modulated by the neighboring VSMCs *in vivo*^25^, we then investigated the paracrine control of ECs by VSMCs under high cyclic stretch.

As shown in Fig. 1d, VSMCs were exposed to different levels of cyclic stretch for 24 h, and the conditioned medium (CM) was used to stimulate ECs for 24 h. The CM from VSMCs exposed to 15% cyclic stretch significantly reduced the expression of GRK6 in ECs (Fig. 1e) and induced EC proliferation analyzed with expression of proliferating cell nuclear antigen (PCNA) (Fig. 1f) and BrdU ELISA (Fig. 1g). In addition, compared with the CM from VSMCs under static condition, the CM from 5%-cyclic-stretch group significantly increased the expression of GRK6 in ECs, while the CM from 15%- group decreased it (Supplementary Fig. S2b). These results indicate that high cyclic stretch applied to VSMCs inhibits expression of GRK6 in ECs in a paracrine manner, which may contribute to the abnormal proliferation of ECs during hypertension.

### VSMC-MPs with 15% cyclic stretch repressed GRK6 expression and promoted EC proliferation

**Figure 2:**
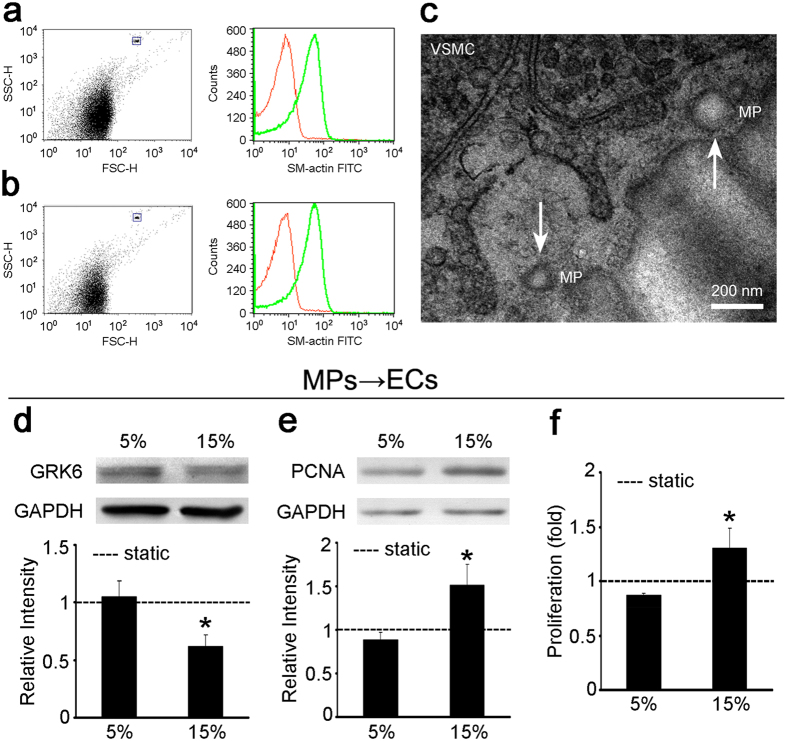
Treatment of ECs with 15%-VSMC-MPs decreased EC GRK6 expression and promoted EC proliferation. (**a**) VSMC-MPs from cultured VSMCs were stained for the VSMC marker SMA and detected by FCM. The dot plot shows the size of the VSMC-MPs, and the histogram shows the expression of SMA in the VSMC-MPs. The red peak in the histogram corresponds to the negative control with an isotype antibody. The blue rectangle shows 2.5–4.5 μm beads. (**b**) VSMC-MPs from the TA were isolated, stained for SMA, and detected by FCM. The dot plot shows the size of the VSMC-MPs, and the histogram shows the expression of SMA in the VSMC-MPs. The red peak in the histogram corresponds to the negative control with an isotype antibody. The blue rectangle shows 2.5–4.5 μm beads. (**c**) VSMC-MPs in the TA were detected by TEM. The white arrow shows the VSMC-MPs. (**d–f**) VSMCs were exposed to static condition, 5% and 15% cyclic stretch for 24 h, respectively, and the VSMC-MPs obtained from each CM were used to stimulate ECs for 24 h. (**d**) GRK6 expression and (**e**) PCNA expression of ECs were detected by western blotting. (**f**) Proliferation of ECs was detected by BrdU ELISA. ‘----’ indicates the static values standardized to 1. The values are shown as the mean ± SD for each condition from at least four independent experiments. *P < 0.05 vs. 5%. Full-length blots are presented in Supplementary Fig. S7.

Recent studies revealed that MPs have crucial roles in intercellular communication^26^; therefore, we investigated the VSMC-derived microparticles (VSMC-MPs) and their roles in the paracrine control of ECs by VSMCs under mechanical cyclic stretch. VSMC-MPs were obtained from CM of VSMCs *in vitro* as described in Methods. For flow cytometry (FCM) analysis, 1.1 μm beads were used to set the gate for MPs and then VSMC-MPs were analyzed. The results showed that, in the culture medium from VSMCs, there were particles with diameters less than 1 μm that expressed the VSMC marker, smooth muscle-specific α-actin (SMA) (Fig. 2a), which suggested the existence of VSMC-MPs *in vitro*.

Then, the existence of VSMC-MPs was verified *in vivo*. The results of FCM analysis revealed the particles with diameters less than 1 μm and expressed SMA (Fig. 2b), suggesting the existence of VSMC-MPs *in vivo*. Transmission electron microscopy (TEM) analysis also showed extracellular particles with a high electron density membrane structure at the media layer of the TA, which were 0.1–1 μm in size (Fig. 2c), confirming the existence of VSMC-MPs *in vivo*.

To detect whether cyclic stretch modulates the release of MPs from VSMCs and to investigate the effect of VSMC-MPs on ECs, VSMCs were exposed to different levels of cyclic stretch for 24 h, then the CM was collected. VSMC-MPs obtained from respective CM were then used to stimulate ECs. Compared with VSMC-MPs treated with 5% cyclic stretch, VSMC-MPs treated with 15% cyclic stretch repressed the expression of GRK6 in ECs (Fig. 2d) and promoted EC proliferation (Fig. 2e and f). In addition, compared with VSMC-MPs from static controls, VSMC-MPs from 5%-cyclic-stretch group had little effect on the expression of GRK6 in ECs, but VSMC-MPs from 15% group significantly reduced GRK6 expression in ECs (Supplementary Fig. S2c). These results indicate that VSMC-MPs participate in the paracrine regulation of ECs by VSMCs under high and pathological cyclic stretch.

### High cyclic stretch induced the secretion of miR-27a via VSMC-MPs

Increasing evidence has indicated that miRs are important components of MPs and, to a large extent, determine their effects^24^,^27^. Therefore, we hypothesized that VSMC-MPs transfer miRs and induce changes in GRK6 expression and proliferation in ECs.

Three prediction websites [miRanda (http://www.microrna.org/microrna/home.do), PicTar (http://pictar.mdc-berlin.de/) and TargetScan (http://www.targetscan.org/)] were used, and four miRs (i.e., miR-19a, miR-19b, miR-27a, and miR-217) were predicted to target the 3′-untranslated region (3′ UTR) of GRK6 according to the score (the sequence comparison is shown in Supplementary Fig. S3a). Mimics of these four miRs were transfected into ECs, and the results showed that miR-27a and miR-217 regulated the expression of GRK6 (Supplementary Fig. S3b). Then, the existence and expression level of miR-27a and miR-217 in VSMC-MPs induced by different cyclic stretch protocols were determined.

**Figure 3:**
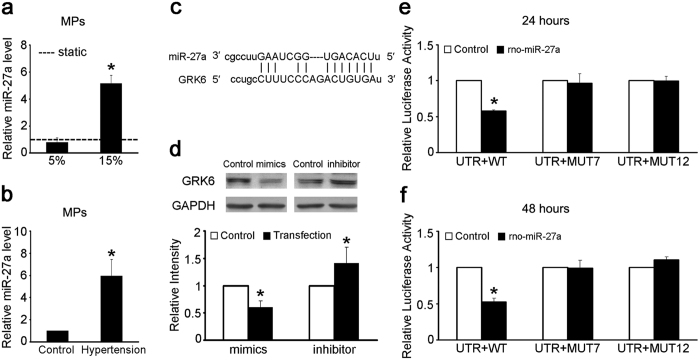
The 15% cyclic stretch treatment increased the miR-27a level in VSMC-MPs, which directly targeted GRK6. (**a**) VSMCs were exposed to static condition, 5% and 15% cyclic stretch for 24 h, respectively, and the VSMC-MPs were obtained from each CM. Level of miR-27a in VSMC-MPs was detected by qPCR. *Caenorhabditis elegans* miR-39 (cel-miR-39) was added to each sample as a spike-in control. ‘----’ indicates the static value standardized to 1. The values are shown as the mean ± SD for each condition from at least four independent experiments. *P < 0.05 vs. 5%. (**b**) Level of miR-27a in VSMC-MPs from the TA isolated from 1-week hypertensive and sham-operated rats was detected by qPCR. Cel-miR-39 was added to each sample as a spike-in control. (**c**) Sequence comparison of miR-27a and the GRK6 3′ UTR. (**d**) The expression of GRK6 in the ECs transfected with miR-27a mimics and inhibitor for 48 h was detected by western blotting. (**e**,**f**) Luciferase activity in the HEK 293 T cells 24 and 48 h after co-transfection with GRK6 WT, MUT7, or MUT12 3′ UTR and the miR-27a mimics compared with co-transfection with NC. The values of control groups were standardized to 1 and the values are shown as the mean ± SD for each condition from at least four independent experiments. *P < 0.05 vs. control. Full-length blots are presented in Supplementary Fig. S7.

Compared with VSMC-MPs from 5% cyclic stretch, the level of miR-27a in VSMC-MPs from 15% cyclic stretch was significantly increased (Fig. 3a), although miR-217 was not detected in VSMC-MPs. In addition, compared with VSMC-MPs under normotensive conditions, the level of miR-27a in VSMC-MPs from the TA isolated from 1-week hypertensive rats was also significantly increased (Fig. 3b). These results indicate that high cyclic stretch caused by hypertension induces the secretion of miR-27a via VSMC-MPs.

The effect of miR-27a on GRK6 was then further confirmed. Figure 3c shows the sequence comparison between miR-27a and the 3′ UTR of GRK6. The role of miR-27a in the repression of GRK6 in ECs was demonstrated by transfecting mimics and inhibitor of miR-27a at a concentration of 100 nmol/L. The miR-27a mimics decreased the expression of GRK6, whereas the inhibitor had the opposite effect (Fig. 3d). The effect of miR-27a was further validated by the response of a GRK6 3′ UTR reporter to the miR-27a mimics in HEK 293 T cells. Co-transfection with the GRK6 wild-type (WT) 3′ UTR and miR-27a mimics for 24 or 48 h led to a significant repression of luciferase activity compared with the co-transfection of GRK6 WT 3′ UTR and miR-27a negative control (NC), but this repression was not observed when the MUT7 (7 sequential bases of the miR-27a seed site mutated) or MUT12 (all 12 bases of the miR-27a seed site mutated) 3′ UTR was transfected (Fig. 3e and f).

These results suggest that miR-27a is transferred from VSMCs to ECs via VSMC-MPs and then decreases the expression of GRK6 in ECs.

### miR-27a was transferred from VSMCs to ECs via VSMC-MPs

To confirm the transfer of miR-27a from VSMCs to ECs, VSMC-MPs from VSMCs exposed to different cyclic stretch protocols were used to treat ECs as described above, and the expression of mature miR-27a and precursor miR-27a (pre-miR-27a) in ECs was detected.

**Figure 4:**
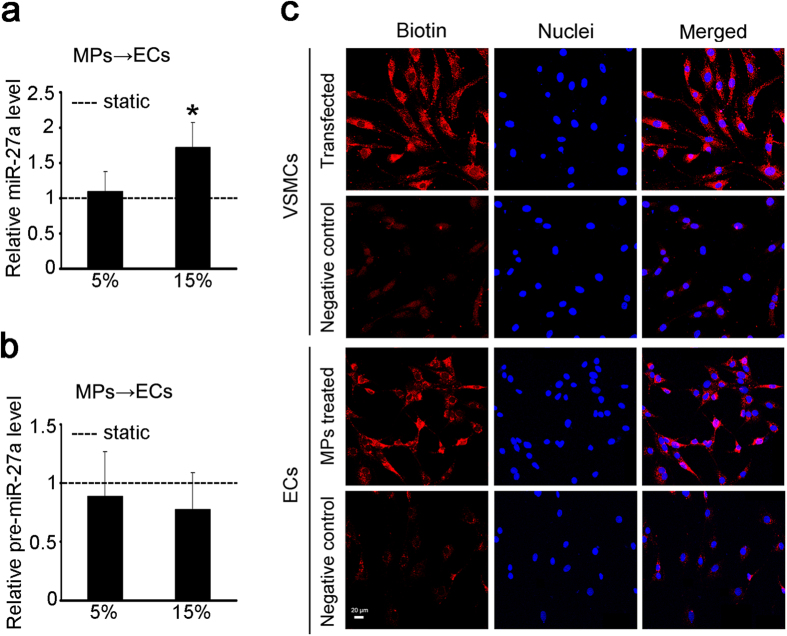
miR-27a was transferred from VSMCs to ECs via VSMC-MPs. (**a**,**b**) VSMCs were exposed to static condition, 5% and 15% cyclic stretch for 24 h, respectively, and the VSMC-MPs obtained from each CM were used to stimulate ECs for 24 h. The expression of miR-27a (**a**) and the expression of pre-miR-27a (**b**) in ECs were detected by qPCR. ‘----’ indicates the static values standardized to 1. The values are shown as the mean ± SD for each condition from at least four independent experiments. *P < 0.05 vs. 5%. (**c**) VSMCs were transfected with B-miR-27a at a concentration of 30 nmol/L for 24 h, and the negative control was transfected with miR-27a without biotin labeling. VSMC-MPs from these VSMCs were isolated to treat ECs. B-miR-27a in the VSMCs and ECs was tracked using rhodamine-labeled streptavidin and detected by immunofluorescence. The six top images show the successful transfection of B-miR-27a in the VSMCs, and the six bottom images show the transfer of miR-27a from VSMCs to ECs. Scale bar = 20 μm.

Compared with VSMC-MPs from 5% cyclic stretch, VSMC-MPs from 15% cyclic stretch showed significantly increased expression of mature miR-27a in the ECs (Fig. 4a), but there was no significant change in the expression of pre-miR-27a (Fig. 4b). These results suggested that the upregulation of miR-27a was the result of the increased exogenous mature miR-27a carried in the VSMC-MPs, rather than of the processing of endogenous precursor miR-27a.

Biotinylated miR-27a (B-miR-27a) was then transfected into VSMCs for miR-27a tracking at a concentration of 30 nmol/L. Treatment of ECs with the VSMC-MPs from VSMCs transfected with B-miR-27a resulted in the presence of B-miR-27a in ECs (Fig. 4c). The presence of B-miR-27a in the cytoplasm of both VSMCs and ECs demonstrated the transmission of miR-27a from VSMCs to ECs via VSMC-MPs.

### The effect of miR-27a and GRK6 on the proliferation of ECs

The above results indicate that high cyclic stretch increases the level of miR-27a in VSMC-MPs, and the miR-27a is then transferred into ECs. Therefore, the effects of miR-27a and GRK6 on EC proliferation were examined.

**Figure 5:**
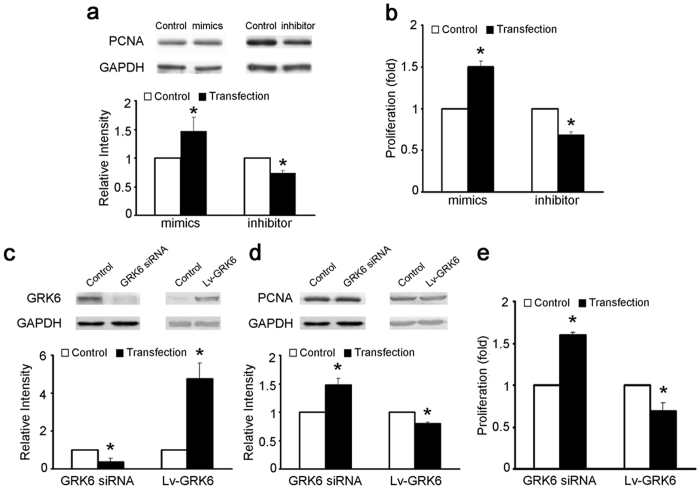
The effect of miR-27a and GRK6 on the proliferation of ECs. (**a**,**b**) ECs were transfected with miR-27a mimics and inhibitor at a concentration of 100 nmol/L for 48 h. (**a**) PCNA expression of ECs was detected by western blotting. (**b**) Proliferation of ECs was detected by BrdU ELISA. (**c–e**) ECs were transfected with GRK6-targeting siRNA at a concentration of 100 nmol/L or Lv-GRK6 for 48 h. (**c**) GRK6 expression and (**d**) PCNA expression of ECs were detected by western blotting. (**e**) Proliferation of ECs was detected by BrdU ELISA. The values of control groups were standardized to 1 and the values are shown as the mean ± SD for each condition from at least four independent experiments. *P < 0.05 vs. control. Full-length blots are presented in Supplementary Fig. S7.

ECs were transfected with miR-27a mimics or inhibitor, and GRK6-targeting siRNA or GRK6 overexpression lentivirus (Lv-GRK6) for 48 h. miR-27a mimics promoted the EC proliferation, whereas the inhibitor reduced EC proliferation (Fig. 5a and b). In addition, GRK6-targeting siRNA suppressed GRK6 expression and promoted EC proliferation, whereas Lv-GRK6 increased GRK6 expression and repressed EC proliferation (Fig. 5c to e). These results indicate a positive effect of miR-27a and a negative effect of GRK6 on EC proliferation.

### Decreasing miR-27a levels *in vivo* attenuated the abnormal EC proliferation during hypertension

**Figure 6:**
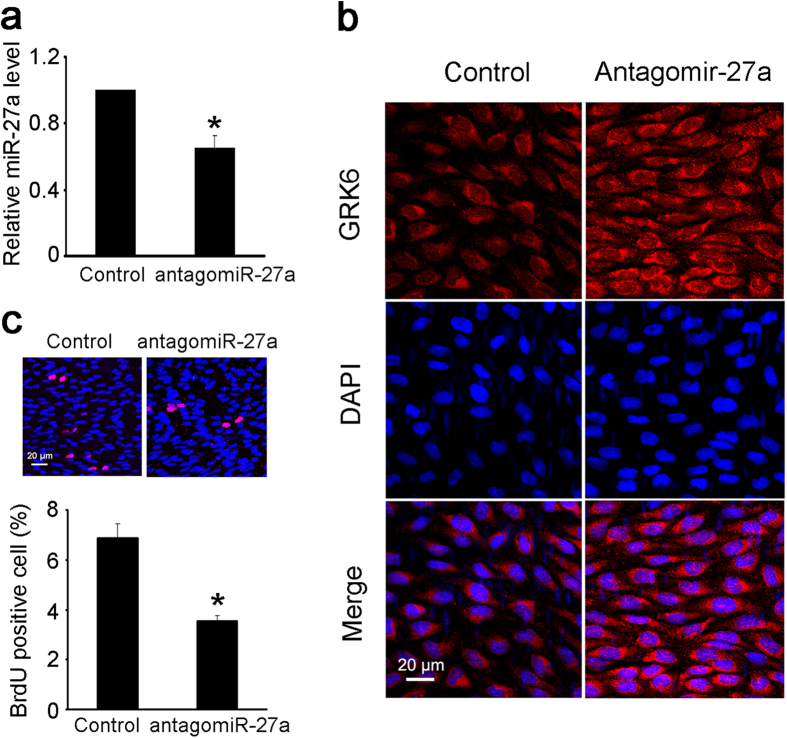
Decreasing the miR-27a level *in vivo* attenuated the abnormal EC proliferation. Perivascular multi-point injection of antagomiR-27a around the left CCA of rats with abdominal aortic coarctation was performed as described, and the right CCA was injected with antagomiR NC locally as a control. Both sides of the CCA were isolated 1 week after surgery for the measurements. (**a**) The level of miR-27a in the left and right CCA was detected by qPCR. (**b**) The expression of GRK6 in ECs from the left and right CCA was detected by immunofluorescence. Scale bar = 20 μm. (**c**) The proliferation of ECs from the left and right CCA was detected by *in situ* BrdU immunofluorescence. The histogram shows the fold change in the level of EC proliferation relative to the control. Scale bar = 20 μm. The values of control groups were standardized to 1. The values are shown as the mean ± SD for each condition from at least four independent experiments. *P < 0.05 vs. control.

Given that the above results indicate that miR-27a has crucial roles in EC proliferation, the potential therapeutic effect of miR-27a on the abnormal EC proliferation that occurs in hypertension was further investigated. An antagomiR-27a was injected around the left CCA, and an antagomiR NC was injected around the right CCA. The antagomiR-27a injection significantly decreased the expression of miR-27a in the left CCA (Fig. 6a) and increased the expression of GRK6 in ECs (Fig. 6b). EC proliferation induced by hypertension was also significantly reversed by the antagomiR-27a injection (Fig. 6c). Furthermore, antagomiR-27a was locally delivered in hypertensive rats for 2 weeks to detect the relative long-term effect *in vivo*. The results revealed that after 2 weeks treatment, antagomiR-27a also significantly decreased EC proliferation in the left CCA (Supplementary Fig. S4). These results demonstrate that locally decreasing miR-27a in the arteries of hypertensive rats attenuates the abnormal proliferation of ECs.

## Discussion

ECs participate in numerous biological processes and have pivotal roles in maintaining vascular homeostasis. The dysfunctions of ECs, such as proliferation, migration, apoptosis, alignment, secretion, and gene expression, are closely related to many vascular disorders^28^. Physiological mechanical stress is essential to maintain EC homeostasis, whereas pathological mechanical stress causes EC dysfunction during hypertension, atherosclerosis, thrombosis, and restenosis^29^. It has been shown that mechanical stimuli modulate EC functions through paracrine regulation by VSMCs. Interestingly, our present results revealed that the effect of different magnitude of cyclic stretch on EC proliferation *in vitro* (Fig. 1g, CM obtained from VSMCs) was modest in comparison with that of hypertension *in vivo* (Fig. 1c). The results suggested that the *in vivo* factors that modulate EC proliferation are multi-faceted and can be very complicated. For example, besides the effect of the neighboring VSMCs and cyclic stretch, hemodynamic forces such as shear stress also regulate the EC proliferation^30^, and various secreted factors from ECs play crucial roles in EC proliferation via both autocrine and paracrine manners^1^. Previous studies demonstrated that TGF-β1 participates in the paracrine control of ECs by VSMCs under low shear stress^31^, and high cyclic stretch induces the expression of Rab28 in ECs through paracrine control by VSMCs via angiotensin II^8^. The present study revealed a novel mechanism by which VSMCs regulate EC functions in response to high cyclic stretch in hypertension: the VSMC-MPs.

A multitude of eukaryotic cells and even prokaryotes have the ability to shed MPs, which are approximately 100–1000 nm in diameter^32^. Given the various types of molecule found on their surface and in their interior, MPs can have crucial roles in cellular communication^26^. Stampfuss *et al*. found that exposure to fluids caused the extremely rapid release of active MP-bound tissue factor from VSMCs when endothelial damage occurred^33^. The study of Essayagh *et al*. demonstrated that MPs from apoptotic VSMCs induced endothelial dysfunction^34^. Recently, increasing evidence has indicated that MPs are a major type of transport for miRs, which determine the effects of MPs^21^,^26^. The present study revealed that high cyclic stretch increased the secretion of miR-27a from VSMCs via VSMC-MPs, and the miRs were subsequently transferred to ECs, where they induced EC proliferation.

miR-27a is an important secreted miR that is involved in many physiological and pathological processes. The findings of Jaiswal *et al*. showed the transmission of miR-27a from the Lucena cell line to the co-cultured recipient cells (breast and lung cancer models) via Lucena-derived MPs^35^. Ovchinnikova *et al*. found that the level of circulating miR-27a was lower in acute heart failure patients with early decreases in renal function^36^. Saha *et al*. demonstrated that miR-27a cargo in monocyte-derived extracellular vesicles from the plasma of alcoholic hepatitis patients can program naive monocytes to polarize into M2 macrophages^37^. The above reports demonstrate the mechanisms or diagnostic significance of secreted miR-27a. Our results revealed not only the mechanism of secreted miR-27a by which hypertension regulates EC functions, but also a novel therapeutic approach to attenuate the abnormal proliferation of ECs in hypertension.

miR-27a/b was also reported to promote angiogenesis^38^. The strain-induced VSMC-MPs modulated tube formation *in vitro* (Supplementary Fig. S5), which suggests that VSMC-MPs from high cyclic stretch may deliver miR-27a and promote EC angiogenesis. However, the sustain elevation of miR-27a associated with VSMC-MPs induced by high cyclic stretch may results in excess angiogenesis, which may be detrimental to hypertension^39^. We also found that GRK6, the direct target of miR-27a, participated in the regulation of secreted miR-27a from VSMCs on the proliferation of ECs in hypertension. To this end, the potential targets of GRK6 that controls EC functions were analyzed by using Ingenuity Pathway Analysis (IPA) software. Such *in silico* analysis revealed that 50 molecules were regulated by GRK6 in which 37 were participated in the control of cell proliferation, and 18 were participated angiogenesis (Supplementary Fig. S6, Supplementary Table S1).

The GRK family contains three subfamilies and seven members: the visual subfamily (GRK1 subfamily), including GRK1 and GRK7, the β-adrenergic receptor kinase family (GRK2 subfamily), including GRK2 and GRK3, and the GRK4 subfamily, including GRK4, GRK5, and GRK6. GRK1 and GRK7 are solely expressed in the retina, and GRK4 is expressed in several organs, such as the kidney and testes, whereas the other four are relatively ubiquitously expressed^40^. The present results suggest a protective role of GRK6 in ECs, and several other studies have also demonstrated a protective effect of GRKs in hypertension. Oliver *et al*. showed that the GRK3 mRNA level in human lymphocytes was inversely related to BP, suggesting a protective effect of GRK3 on the regulation of BP^10^. Tutunea-Fatan *et al*. revealed that genetic knockdown of GRK2 using a small hairpin RNA in mice caused spontaneous hypertension between 8 and 12 weeks of age^9^. However, the physiological effects of GRKs in hypertension are complicated, and there are differing reports. Izzo *et al*. found that hypertensive patients and spontaneously hypertensive rats had increased expression of GRK2 in their lymphocytes and VSMCs^41^. The data of Keys *et al*. suggested that VSMC-specific overexpression of GRK5 elevated BP in both male and female mice^42^.

The differing effects of GRKs on hypertension might result from the various causes of hypertension (i.e., different hypertensive models) and the diverse roles of the various GRK subtypes in different tissues. It has been reported that GRK6 deficiency in mice decreased chemotaxis in the lymphocytes but increased chemotaxis in polymorphonuclear cells^43^. Another reason for these differing effects is the diverse functional mechanisms of GRKs. They can phosphorylate both G-protein-coupled receptors (GPCRs) and non-GPCRs in phosphorylation-dependent and phosphorylation-independent manners (direct binding), and can also phosphorylate both active and inactive GPCRs^44^.

In addition, recent research reported the existence of stem cell derived vascular progeny which may play important role in vascular diseases^45^,^46^. Our results also revealed the existence of the stem cell markers including nestin and SOX10 in primary VSMCs obtained by the explant technique (data not shown). Whether the stem cell-derived vascular progenies release MPs and the potential roles of these cells in vascular remodeling during hypertension still need future study using lineage tracing experiments *in vivo*.

**Figure 7:**
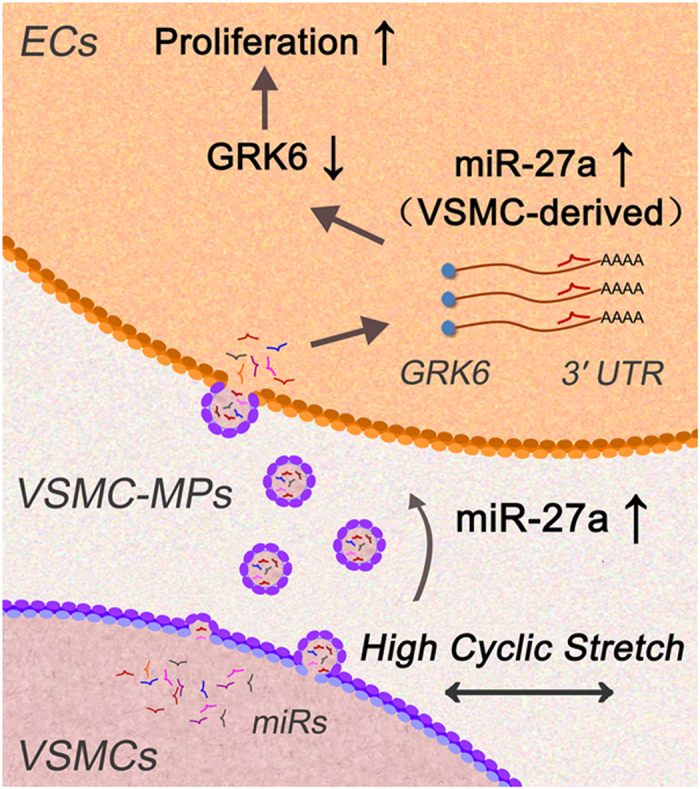
Schematic drawing of the role of secreted miR-27a in abnormal EC proliferation during hypertension. Pathologically elevated cyclic stretch increased the secretion of miR-27a, which was transferred from VSMCs to ECs via the VSMC-MPs, subsequently targeted GRK6, and induced EC proliferation.

To conclude, as shown in Fig. 7, the present study revealed that pathologically increased cyclic stretch increased the secretion of miR-27a from VSMCs via VSMC-MPs. The miRs subsequently transferred to ECs, where they targeted GRK6 and finally induced EC proliferation. Therefore, locally decreasing miR-27a could be a novel therapeutic approach to attenuate the abnormal proliferation of ECs that occurs in hypertension.

## Methods

### Ethics approval

The animal care and experimental protocols were in accordance with the Animal Management Rules of China (Documentation 55, 2001, Ministry of Health, China), and the study was approved by the Animal Research Committee of Shanghai Jiao Tong University.

### Hypertensive rat model

The hypertensive rat model was established by abdominal aortic coarctation^27^,^47^. Male Sprague-Dawley rats weighing 200 ± 15 g were anesthetized with isoflurane inhalation and treated under sterile conditions. The abdominal aorta was exposed, and a piece of surgical silk suture (3-0) was used to surround the aorta near the bifurcation to the right kidney. A needle with an outer diameter of 0.9 mm was placed in parallel to the aortic segment. After tying the needle and aortic segment together with the suture, the needle was retrieved immediately, leaving a lumen with a diameter of 0.9 mm at the constriction site; the opening was then closed. Sham-operated rats were treated with the same procedure except there was no constriction of the aorta. BP was measured through a catheter introduced into one of the carotid arteries^20^.

### Cell culture

Primary rat aortic ECs were obtained by the digestive method, cultured in endothelial cell basal medium-2 (Lonza) and characterized by the EC marker von Willebrand factor (DAKO)^48^. Primary rat aortic VSMCs were obtained by the explant technique, cultured in Dulbecco’s modified eagle medium (Gibco) supplemented with 10% FBS and characterized by the VSMC marker SMA (DAKO)^49^. ECs at passages 2–4 and VSMCs at passages 4–7 were used.

### Cyclic stretch application

Cells were seeded on flexible silicone-bottom plates (Flexcell International) at a density of 3 × 10^5^ cells per well. Twenty-four hours later, the culture medium was replaced with serum-free medium for another 24 h to synchronize the cells. Cells in the serum-free medium were then exposed to cyclic stretch provided by the FX-4000T Strain Unit (Flexcell International) with an elongation magnitude of either 5% or 15%, at a consistent frequency of 1.25 Hz.

### VSMC-MP extraction

To obtain VSMC-MPs from cultured cells *in vitro*, the culture medium of VSMCs was collected and centrifuged at 1,500 g for 15 min to remove cell debris and then at 12,500 g for 5 min to remove apoptotic bodies. The supernatant was centrifuged at 20,500 g for 90 min to pellet the VSMC-MPs^50^,^21^. The pellets were then washed with sterile PBS and pelleted again at 20,500 g for 90 min. All centrifugations were performed at 4 °C.

To obtain VSMC-MPs from the arteries *in vivo*, the TA was first minced in filtered DMEM, and a similar centrifugation procedure to that described above was then performed^21^.

Pelleted VSMC-MPs were resuspended in DMEM and used while fresh.

### Flow cytometry analysis

VSMC-MPs were characterized by BD FACSCalibur using SMA-FITC. After 4% paraformaldehyde fixation and 0.1% Triton X-100 permeabilization, SMA was detected with mouse anti-SMA-FITC (Sigma-Aldrich) and an anti-mouse IgG2α-FITC isotype control (Sigma-Aldrich). To evaluate the size and set the gate for VSMC-MPs, polystyrene latex beads (1.1 μm, Sigma-Aldrich) and Nile red fluorescent particles 2.5–4.5 μm (BD) were used as reference beads.

### Transmission electron microscopy

Vascular rings were fixed with 2.5% glutaraldehyde and 1% osmic acid, dehydrated with 50%, 70%, 90% ethanol solution, 90% ethanol, and 90% acetone solution (1:1), and then embedded in Epon-Araldite. Fractions stained with uranyl acetate were examined using a JEM-2010 (Oxford) TEM.

### Cell proliferation assay

For *in situ* BrdU immunofluorescence, EC proliferation was analyzed using the *In Situ* Cell Proliferation Kit, FLUOS (Roche). Twenty-four hours before harvest, BrdU (Sigma-Aldrich) was intraperitoneally injected into rats (200 mg/kg). After 24 h, the TA and CCA were isolated, fixed, and dehydrated with isopentane. The stretched preparations were subsequently rehydrated with PBS, fixed with 70% ethanol in 50 mmol/L glycine buffer, digested with 0.05% trypsin solution, denatured with 2 mol/L HCL, and labeled with anti-BrdU antibody (1:800, Sigma-Aldrich) and a fluorescent secondary antibody. Nuclei were stained with DAPI. After mounting, EC proliferation was visualized using an Olympus FV1000 laser scanning confocal microscope.

For the BrdU ELISA, EC proliferation was analyzed using a colorimetric BrdU kit (Roche). Eight hours before detection, the BrdU labeling reagent was added to the culture medium (1:1000). ECs were fixed and labeled according to the manufacturer’s instructions. The absorbance at 450 nm was measured in an ELISA plate reader (Bio-Rad 680).

### Immunofluorescence

After whole-body perfusion with 4% paraformaldehyde, the TA and CCA were isolated and fixed with 4% paraformaldehyde. The stretched preparations were subsequently permeabilized with 0.3% Triton X-100, blocked by 5% BSA, and incubated with a primary antibody and then a fluorescent secondary antibody. Nuclei were stained with DAPI. After mounting, the slips were visualized using an Olympus FV1000 laser scanning confocal microscope.

### RNA transfection

The sequences of the rno-miR-27a mimics were: 5′-UUC ACA GUG GCU AAG UUC CGC-3′ and 5′-GGA ACU UAG CCA CUG UGA AUU-3′; rno-miR-19a mimics were: 5′-UGU GCA AAU CUA UGC AAA ACU GA-3′ and 5′-AGU UUU GCA UAG AUU UGC ACA UU-3′; rno-miR-19b mimics were: 5′-UGU GCA AAU CCA UGC AAA ACU GA-3′ and 5′-AGU UUU GCA UGG AUU UGC ACA UU-3′; rno-miR-217 mimics were: 5′-UAC UGC AUC AGG AAC UGA CUG-3′ and 5′-AGU CAG UUC CUG AUG CAG UAU U-3′. The sequence of the rno-miR-27a inhibitor was 5′-GCG GAA CUU AGC CAC UGU GAA-3′. The sequences of the rat GRK6 siRNA were: 5′-CCA CAG ACC AAG ACU UCU ATT-3′ and 5′-UAG AAG UCU UGG UCU GUG GTT-3′. The sequence of the NC had no homology to rat genes. ECs were transfected with mimics, inhibitor, and siRNA using Lipofectamine^TM^ 2000 (Invitrogen) according to the manufacturer’s instruction at a concentration of 100 nmol/L.

### Lentivirus infection

Lv-GRK6 and scrambled control (GFP lentiviral vector) were synthesized by GenePharma (Shanghai), with the concentrations of approximately 10^8^ TU/ml. ECs were seeded on 6-well plates at a density of 2 × 10^5^ cells per well or seeded on 96-well plates at a density of 1 × 10^4^ cells per well overnight, and then infected with lentivirus and Polybrene (10:1).

### qPCR

Total RNA was isolated using TRIzol reagent (Invitrogen). For the isolation of RNAs from VSMC-MPs, *Caenorhabditis elegans* miR-39 (cel-miR-39) was added to each sample as a spike-in control. Isolated RNAs were reversed transcribed into complementary DNA with the M-MLV RT system using the RT primer for the miRs (GenePharma) or olig dT for the mRNA. qPCR was performed with the SYBR Green Supermix (TaKaRa), and the levels of precursor and mature miRs were normalized against the control U6 snRNA or cel-miR-39. The level of GRK6 was normalized against GAPDH. Specific primers for rat GRK6 were: sense 5′-TAC CTA AGC ATG GCC CCT T-3′, and anti-sense 5′-TCG GTA CTG CCT AAA GGT GT-3′. Specific primers for rat GAPDH were: sense 5′-TGA AGG GTG GGG CCA AAA-3′, and anti-sense 5′-GCT GAC AAT CTT GAG GGA GT-3′.

### Dual luciferase reporter assay

For luciferase reporter experiments, one 60-bp WT oligo from positions 2601–2660 (including the miR-27a seeding site) of the GRK6 3′ UTR or two mutated (MUT) oligos (MUT12 and MUT7) were obtained by gene synthesis (Supplementary Table S2) and inserted downstream of the luciferase reporter gene (psiCHECK-2, Promega). HEK-293T cells were transfected with these reporter plasmids (WT, MUT7, and MUT12) together with the miR-27a mimics or NC using Lipofectamine^TM^ 2000 (Invitrogen). Twenty-four and 48 h later, firefly and *Renilla* luciferase activities were measured consecutively using the dual luciferase reporter assay system (Promega).

### miR-27a tracing

Biotinylated miR-27a (B-miR-27a, synthesized by RiboBio) was transfected into VSMCs using Lipofectamine^TM^ 2000 (Invitrogen) at a concentration of 30 nmol/L, and the culture media was exchanged 6–8 h after transfection to remove the free B-miR-27a. The culture media was collected 24 h later, and the VSMC-MPs were isolated to treat ECs. Staining of B-miR-27a in ECs and VSMCs was performed using rhodamine-labeled streptavidin (KPL).

### Western blotting

Proteins (30 μg) were electrophoretically separated with 12% SDS-PAGE and transferred to nitrocellulose membranes (Hybond, Amersham). Western blotting was performed using antibodies directed against GRK6 (1:500, Santa Cruz Technologies), PCNA (1:500, Proteintech), and GAPDH (1:1000, Proteintech). After incubation with alkaline phosphatase-conjugated secondary antibodies (Jackson Immunoresearch), the signals were detected by nitroblue tetrazolium-bromochloroindolyl phosphate (Bio Basic Inc.) and quantified by Quantity One software (Bio-Rad).

### Local injection of antagomiR-27a

AntagomiR-27a and antagomiR NC were synthesized by GenePharma (Shanghai). The left carotid artery of hypertensive rats with abdominal aortic coarctation was injected with antagomiR-27a via a perivascular multi-point injection. The right carotid artery was treated with the scrambled antagomiR NC as a control.

AntagomiR-27a and antagomiR NC (20 nmol) were first injected 1 day after the abdominal aortic coarctation surgery and then another 10 nmol was injected every 3 days ever after. Both sides of the CCAs were isolated 7 or 14 days after surgery for measurements^51^,^52^.

### Statistical analysis

Each experiment was performed at least four times, and all values are expressed as the mean ± *SD*. One-way ANOVA was used to compare the results between two groups. Values of *P* < 0.05 were considered significant.

## Additional Information

**How to cite this article**: Wang, L. *et al*. Secreted miR-27a Induced by Cyclic Stretch Modulates the Proliferation of Endothelial Cells in Hypertension via GRK6. *Sci. Rep.* **7**, 41058; doi: 10.1038/srep41058 (2017).

**Publisher's note:** Springer Nature remains neutral with regard to jurisdictional claims in published maps and institutional affiliations.

## Supplementary Material

Supplementary Figures and Tables
