# Supplementary material for: Secreted miR-27a Induced by Cyclic Stretch Modulates the Proliferation of Endothelial Cells in Hypertension via GRK6
Source: Sci Rep. 2017 Jan 20;7:41058. doi: 10.1038/srep41058 (PMC5247685; doi:10.1038/srep41058)
Supplement: Supplementary Figures and Tables [file srep41058-s1.doc]

**Secreted miR-27a Induced by Cyclic Stretch Modulates the Proliferation of Endothelial Cells in Hypertension via GRK6.**

**Lu Wang^1^, Han Bao^1^, Kai-Xuan Wang^1^, Ping Zhang^1^, Qing-Ping Yao^1^, Xiao-Hu Chen^1^, Kai Huang^1^, Ying-Xin Qi^1^, Zong-Lai Jiang^1^**

*1. Institute of Mechanobiology & Medical Engineering, School of Life Sciences & Biotechnology, Shanghai Jiao Tong University, Shanghai, China*

**Supplemental Method**

***In vitro* tube formation assay**

Matrigel Matrix (CORNING) were added into 24-well plates (400 μL per well) and incubated in a CO_2_-free incubator at 37 °C for 30 min. ECs were seeded at a density of 2 × 10^4^ cells per well. VSMCs were exposed to 5% or 15% cyclic stretch for 24 h, and the VSMC-MPs obtained from each CM were used to stimulate ECs for 6 h. The images of four representative fields were photographed (IX-71, Olympus), and the averages of the total length of complete tubes per unit area among groups were measured by using Image Pro-Plus 6.3 software.

**Supplemental Figures**

**Supplemental Figure S1**


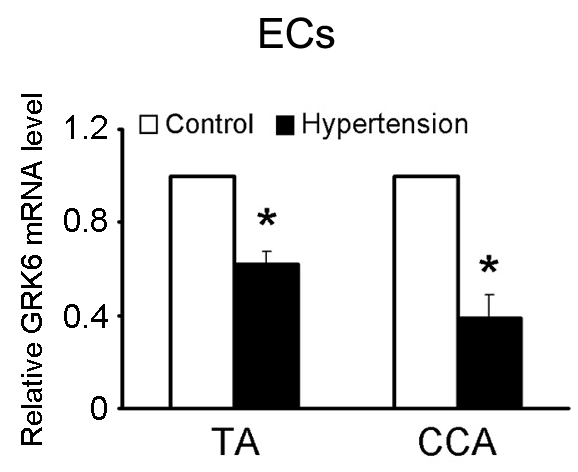


**Figure S1.** Hypertension decreased GRK6 mRNA level in ECs. The GRK6 mRNA level in the ECs of the thoracic aorta (TA) and common carotid artery (CCA) from hypertensive rats and controls were detected by qPCR. Values are shown as mean ± SD for each condition from five independent experiments. **P*<0.05 vs. control.

**Supplemental Figure S2**

**
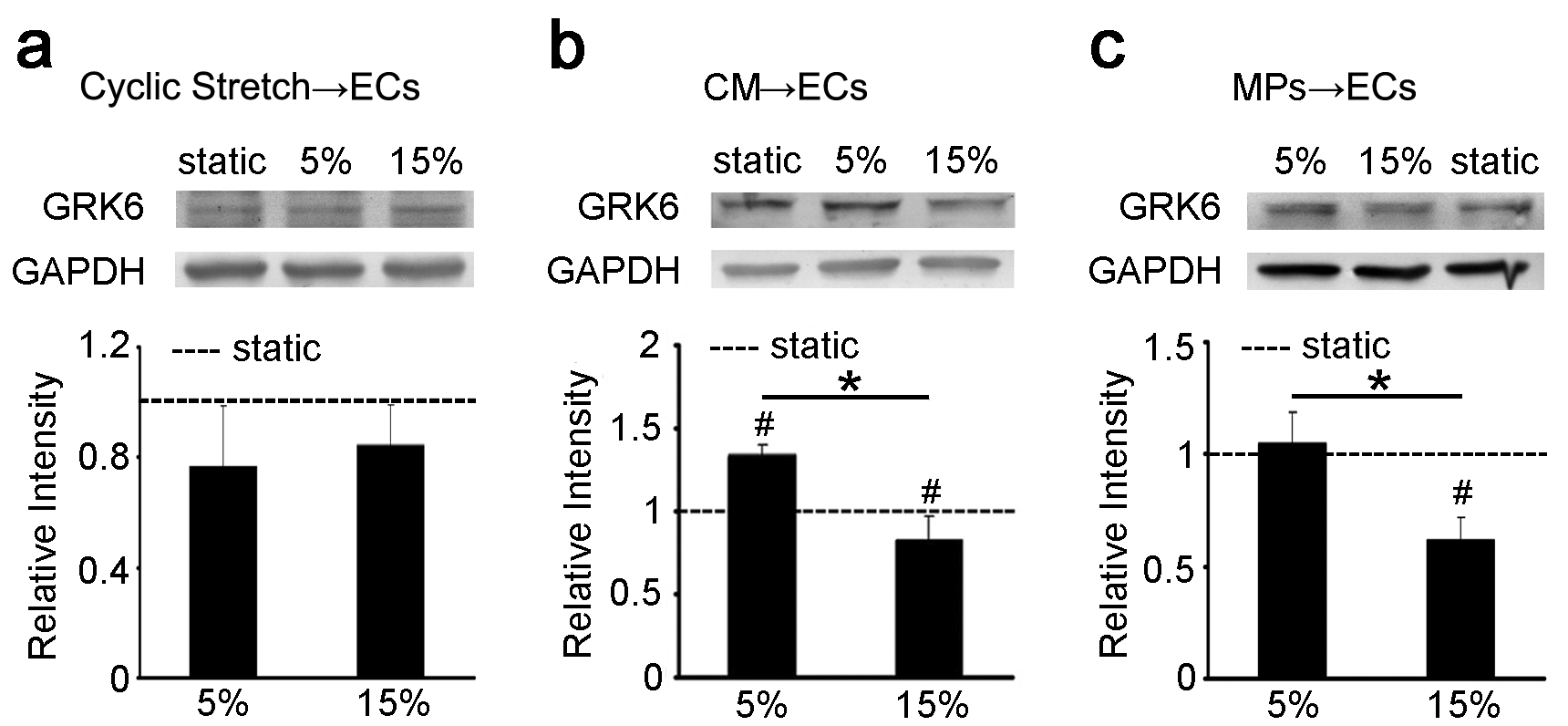
**

**Figure S2.** The effects of different cyclic stretch on expression of GRK6 in ECs. **(a)** Direct effect of different cyclic stretch on GRK6 expression in ECs. ECs were exposed to static condition, 5% and 15% cyclic stretch for 24 h, respectively. **(b)** Effect of condition medium (CM) from VSMCs exposed to different cyclic stretch on GRK6 expression in ECs. VSMCs were exposed to static condition, 5% and 15% cyclic stretch for 24 h, respectively, and the CM was used to stimulate ECs for other 24 h. **(c)** Effect of VSMC derived microparticles (VSMC-MPs) from CM treated with different cyclic stretch on GRK6 expression in ECs. VSMCs were exposed to static condition, 5% and 15% cyclic stretch for 24 h, respectively, and the VSMC-MPs obtained from each CM were used to stimulate ECs for other 24 h. The expression of GRK6 in ECs was detected by western blotting. ‘----’ indicates the static value standardized to 1. The values are shown as the mean ± SD for each condition from at least four independent experiments. **P*<0.05 vs. 5%, ^#^*P*<0.05 vs. static. Full-length blots are presented in [Supplementary Fig.](http://www.nature.com/articles/srep28218#s1)S7.

**Supplemental Figure S3**


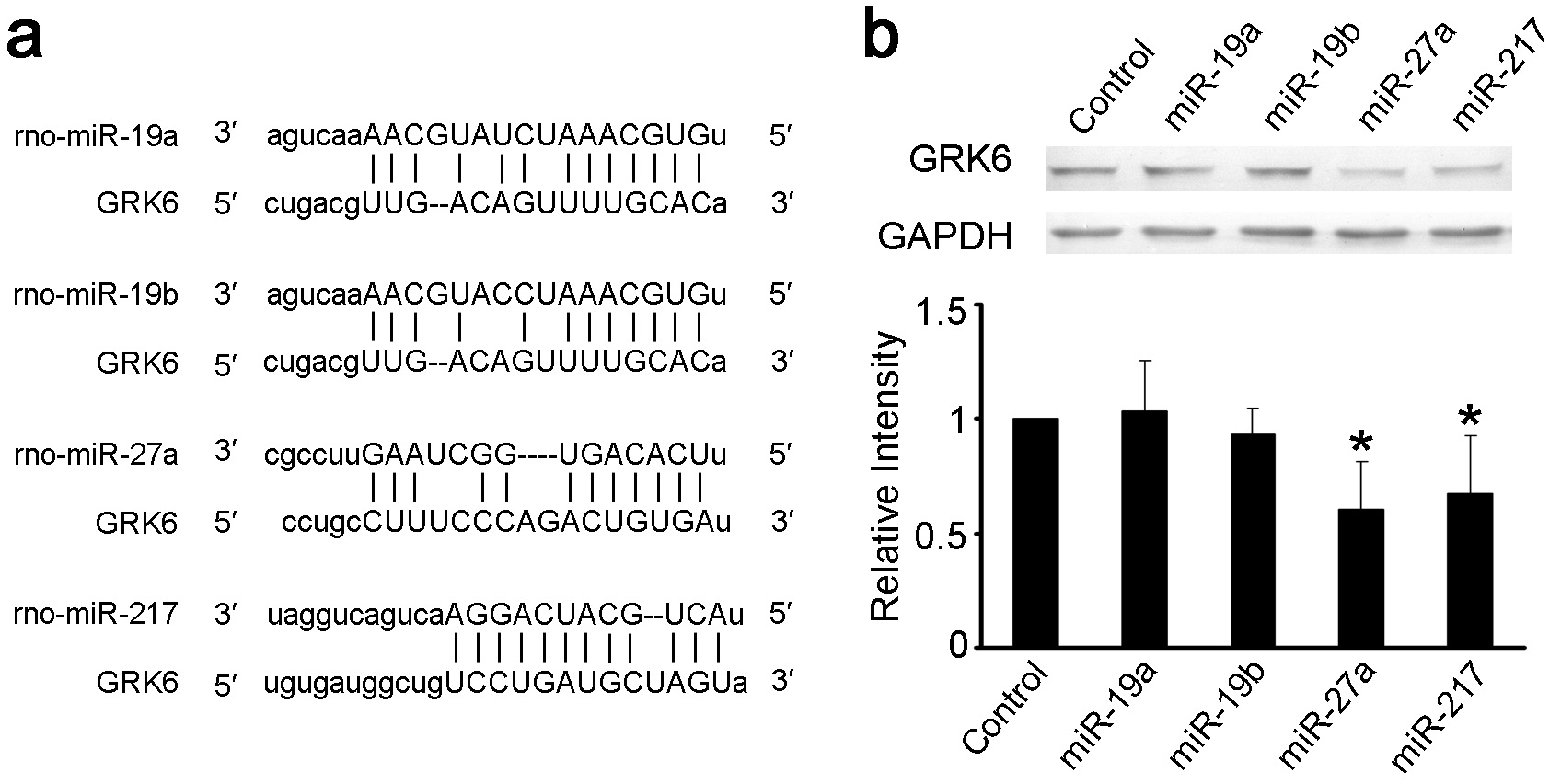


**Figure S3.** Effect of miR-19a, miR-19b, miR-27a, and miR-217 mimics on the expression of GRK6 in ECs. **(a)** Sequence comparison of miR-19a, miR-19b, miR-27a, miR-217, and the GRK6 3′ UTR. **(b)** ECs were transfected with the mimics of the four miRs at a concentration of 100 nmol/L, and the expression of GRK6 in the ECs was detected by western blotting. The value of control group was standardized to 1 and the values are shown as the mean ± SD for each condition from at least four independent experiments. **P* <0.05 vs*.* control. Full-length blots are presented in [Supplementary Fig.](http://www.nature.com/articles/srep28218#s1)S7.

**Supplemental Figure S4**


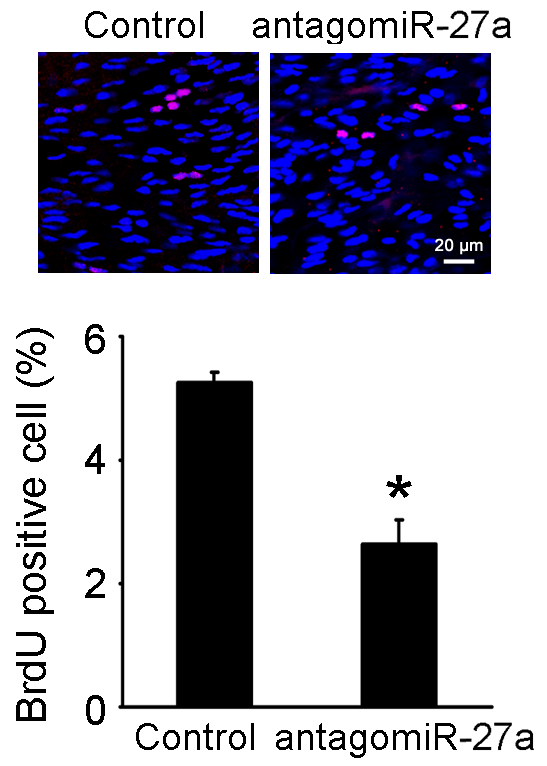


**Figure S4.** Local injection of antagomiR-27a for 2 weeks significantly decreased EC proliferation *in vivo*. The proliferation of ECs from the left and right CCA was detected by *in situ* BrdU immunofluorescence. The histogram shows the fold change of EC proliferation relative to the control. Scale bar = 20 μm. The values are shown as the mean ± SD for each condition from four independent experiments. **P*<0.05 vs. control.

**Supplemental Figure S5**


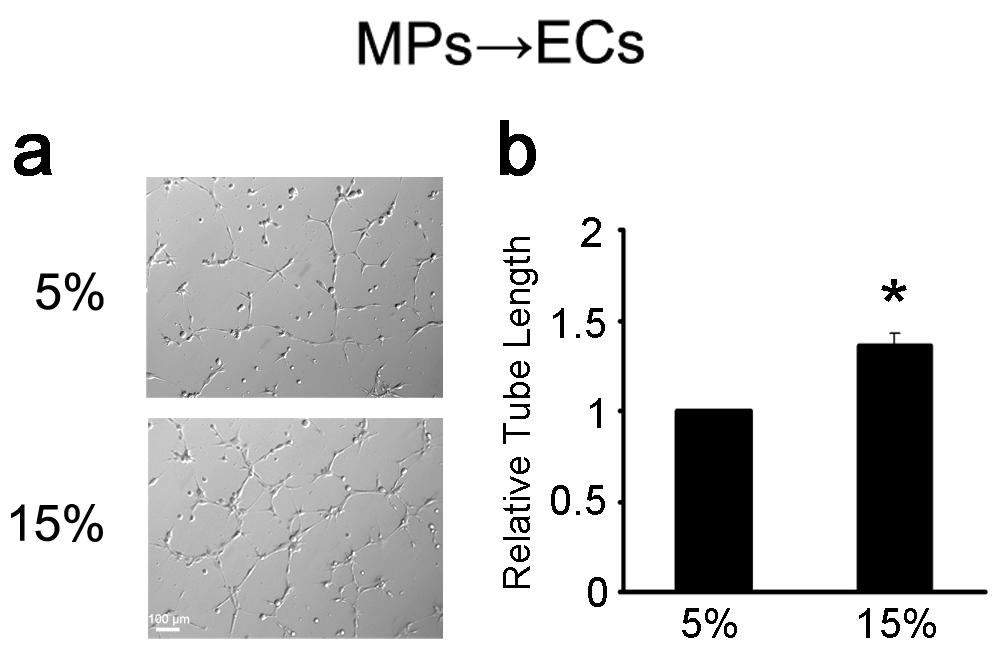


**Figure S5.** The effect of VSMC-MPs from different cyclic stretch on tube formation of ECs. VSMCs were exposed to 5% or 15% cyclic stretch for 24 h, and the VSMC-MPs obtained from condition medium were used to stimulate ECs for 6 h. **(a)** Tube formation of ECs under different conditions. Scale bar = 100 μm. **(b)** Quantification of tube length by Image Pro-Plus. The values are shown as the mean ± SD for each condition from three independent experiments. **P*<0.05 vs. 5%.

**Supplemental Figure S6**


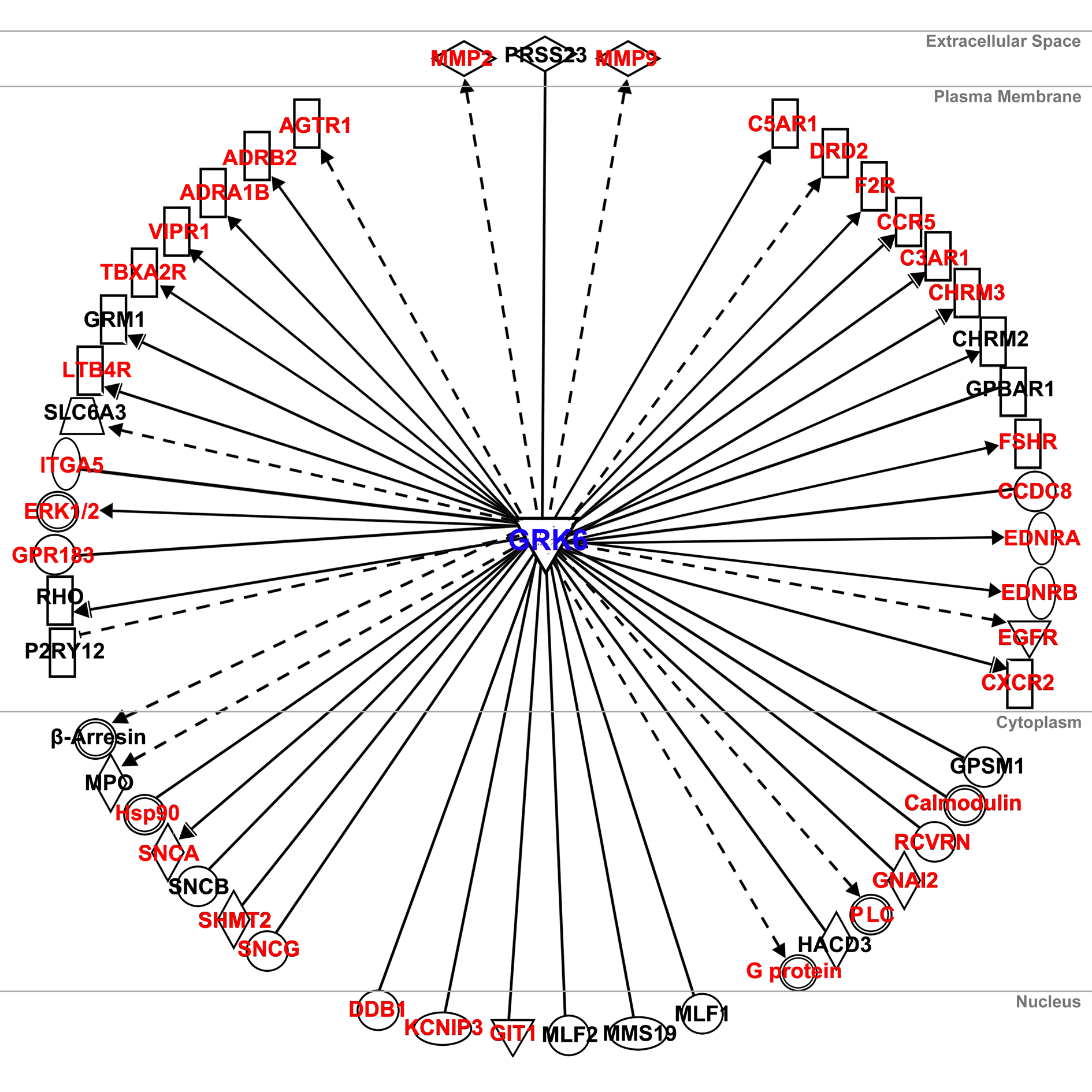


**Figure S6.** The target molecules of GRK6 analyzed by using Ingenuity Pathway Analysis (IPA) software. The target molecules participated in the regulation of cell proliferation were marked red.

**Supplemental Figure S7**


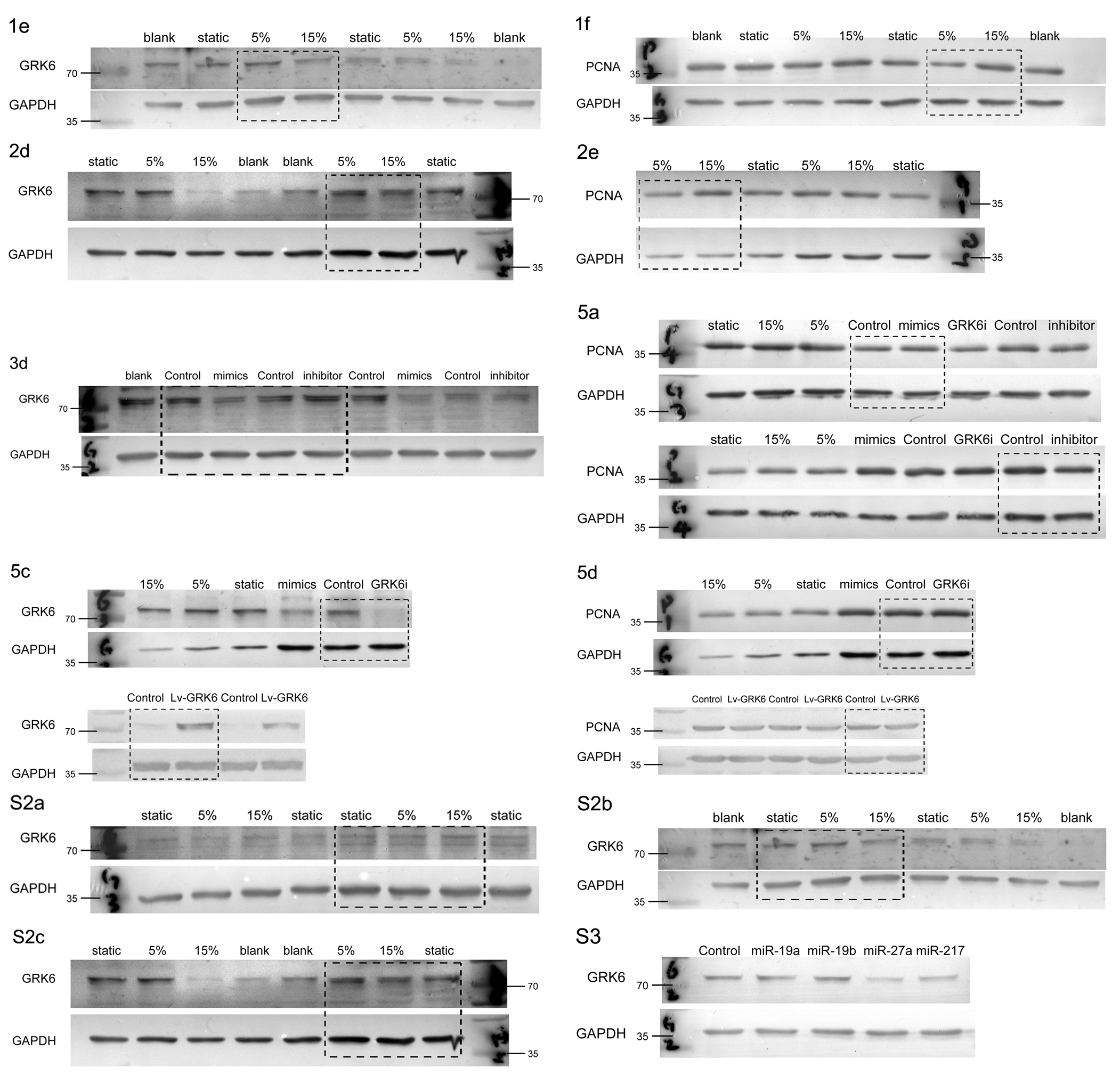


**Figure S7.** The full-length blots of each cropped western blotting in Figures.

**Supplemental Tables**

**Supplemental Table S1.** Functional classification of the target molecules of GRK6 analyzed by Ingenuity Pathway Analysis (IPA).

| **Function** | **Molecule** |
| --- | --- |
| **Angiogenesis** | ADRB2, AGTR1, C3AR1, C5AR1, CCR5, CXCR2, DRD2, EDNRA, EDNRB, EGFR, ERK1/2, F2R, FSHR, ITGA5, LTB4R, MMP2, MMP9, TBXA2R |
| **Apoptosis** | ADRA1B, ADRB2, AGTR1, C3AR1, C5AR1, Calmodulin, CCDC8, CCR5, CXCR2, DDB1, DRD2, EDNRA, EDNRB, EGFR, ERK1/2, F2R, FSHR, GNAI2, GRM1, Hsp90, HSP90AA1, HSP90AB1, ITGA5, KCNIP3, MMP2, MMP9, MPO, RHO, SNCA, SNCB, SNCG, TBXA2R, VIPR1 |
| **Binding** | Calmodulin, CCR5, CXCR2, EGFR, ERK1/2, F2R, Hsp90, ITGA5, MMP2, MPO, P2RY12, PLC, SLC6A3, VIPR1 |
| **Cell death** | ADRA1B, ADRB2, AGTR1, C3AR1, C5AR1, Calmodulin, CCDC8, CCR5, CXCR2, DDB1, DRD2, EDNRA, EDNRB, EGFR, ERK1/2, F2R, FSHR, GNAI2, GRM1, Hsp90, HSP90AA1, HSP90AB1, ITGA5, KCNIP3, MMP2, MMP9, MPO, P2RY12, PLC, RHO, SLC6A3, SNCA, SNCB, SNCG, TBXA2R, VIPR1 |
| **Differentiation** | ADRB2, C3AR1, C5AR1, CCR5, CXCR2, DRD2, EDNRA, EDNRB, EGFR, ERK1/2, F2R, FSHR, G protein, GIT1, HSP90AA1, HSP90AB1, ITGA5, MLF1, MMP2, MMP9, P2RY12, PLC, SNCA |
| **Endocytosis** | ADRB2, CCR5, EGFR, HSP90AA1, MMP9, PLC, SNCA, SNCB, SNCG |
| **Homing** | AGTR1, C3AR1, C5AR1, CCR5, CXCR2, DRD2, EDNRA, EDNRB, EGFR, ERK1/2, F2R, GIT1, GNAI2, GPR183, GPSM1, ITGA5, LTB4R, MMP2, MMP9, PLC, VIPR1 |
| **Migration** | ADRB2, Beta Arrestin, C3AR1, C5AR1, Calmodulin, CCR5, CHRM3, CXCR2, DRD2, EDNRA, EDNRB, EGFR, ERK1/2, F2R, G protein, GIT1, GNAI2, GPR183, GPSM1, Hsp90, HSP90AA1, HSP90AB1, ITGA5, LTB4R, MMP2, MMP9, MPO, P2RY12, PLC, RHO, SNCA, SNCG, TBXA2R, VIPR1 |
| **Proliferation** | ADRA1B, ADRB2, AGTR1, C3AR1, C5AR1, Calmodulin, CCDC8, CCR5, CHRM3, CXCR2, DDB1, DRD2, EDNRA, EDNRB, EGFR, ERK1/2, F2R, FSHR, G protein, GIT1, GNAI2, GPR183, Hsp90, HSP90AA1, HSP90AB1, ITGA5, KCNIP3, LTB4R, MMP2, MMP9, PLC, RCVRN, SHMT2, SNCA, SNCG, TBXA2R, VIPR1 |

**Supplemental Table S2.** The sequences of the GRK6 wild-type (WT) 3′ UTR and GRK6 mutated (MUT) 3′ UTR. The differences between WT and MUT are shown in red.

|  | **Mutation Sites** |
| --- | --- |
| **WT**  **MUT7**  **MUT12** | …cctagcCTTtcCCagACTGTGAt…  …cctagcCTTtcCCagTGACACTt…  …cctagcGAAtcGGagTGACACTt… |
